# Risk Factors in Third and Fourth Degree Perineal Tears in Women in a Tertiary Centre: An Observational Ambispective Cohort Study

**DOI:** 10.3390/jpm11080685

**Published:** 2021-07-21

**Authors:** Juan A. Barca, Coral Bravo, Maria P. Pintado-Recarte, Ignacio Cueto-Hernández, Javier Ruiz-Labarta, Yolanda Cuñarro, Julia Buján, Melchor Alvarez-Mon, Miguel A. Ortega, Juan A. De León-Luis

**Affiliations:** 1 Department of Public and Maternal and Child Health, School of Medicine, Complutense University of Madrid, 28040 Madrid, Spain; barcajuanantonio@gmail.com (J.A.B.); cbravoarribas@gmail.com (C.B.); ppintadorec@yahoo.es (M.P.P.-R.); ignaciocuetohernandez@gmail.com (I.C.-H.); javruila@hotmail.com (J.R.-L.); yolanda.cunarro@gmail.com (Y.C.); jaleon@ucm.es (J.A.D.L.-L.); 2 Department of Obstetrics and Gynecology, University Hospital Gregorio Marañón, 28009 Madrid, Spain; 3 Health Research Institute Gregorio Marañón, 28009 Madrid, Spain; 4 Department of Medicine and Medical Specialties, Faculty of Medicine and Health Sciences, University of Alcalá, 28801 Alcalá de Henares, Madrid, Spain; mjulia.bujan@uah.es (J.B.); mademons@gmail.com (M.A.-M.); 5 Ramón y Cajal Institute of Sanitary Research (IRYCIS), 28034 Madrid, Spain; 6 Immune System Diseases-Rheumatology and Internal Medicine Service, Center for Biomedical Research Network for Liver and Digestive Diseases (CIBEREHD), University Hospital Príncipe de Asturias, 28801 Alcalá de Henares, Madrid, Spain

**Keywords:** risk factors, perineal tear, obstetric vaginal tear, obstetric injuries

## Abstract

Objectives: To analyze the main risk factors associated with third and fourth degree postpartum perineal tears in women attended to in our obstetrics service. Methods: An observational, retrospective, hospital cohort study was carried out in women whose deliveries were attended to in the obstetrics service of the Hospital General Universitario Gregorio Marañón de Madrid (HGUGM), during the period from January 2010 to April 2017. Results: During the study period, a total of 33,026 patients were included in the study. For maternal variables, the associated increased risk of severe perineal tearing in nulliparous women is OR = 3.48, for induced labor OR = 1.29, and for instrumental delivery by forceps OR = 4.52 or spatulas OR = 4.35; for the obstetric variable of episiotomy, it is OR = 3.41. For the neonatal variables, the weight of the newborns has a directly proportional relationship with the risk of severe tears, and for birth weights of 3000 g (OR = 2.41), 3500 g (OR = 1.97), and 4000 g (OR = 2.17), statistically significant differences were found in each of the groups (*p* < 0.05). Conclusion: Primiparity, induction of labor, episiotomy, instrumental delivery with forceps or spatula, and a birth weight of 3000 g or more are significantly associated with an increased risk of third and fourth degree perineal tears.

## 1. Introduction

In recent years, the relationship between vaginal birth and pelvic floor diseases has been studied. These diseases are often due to pelvic floor trauma and can be objectified as perineal tears after childbirth; they are categorized into different levels of injury depending on the involvement of the anatomical structures [1,2].

Among those with the highest level of involvement are third degree perineal tears (which involve injury to the external and internal anal sphincter) and fourth degree perineal tears (which involve injury to both external and internal sphincters and the rectal mucosa). In the literature, we can find a wide range of involvement, depending on the studies, with an incidence between 0.1% and 10.9% among women who have a vaginal delivery [3,4]. The most common pelvic floor dysfunctions associated with this type of injury are urinary incontinence (UI), fecal/anal incontinence (FI/AI), chronic pelvic pain, and sexual dysfunctions [5].

The risk factors associated with the development of third and fourth degree tears include maternal characteristics such as age, weight gain during pregnancy, parity; neonatal variables such as birth weight; and obstetric or intrapartum variables such as induction of labor, gestational age, type of anesthesia, type of delivery, and including instrumental deliveries [6,7,8,9,10], often these are interrelated variables.

Within the group of births that end vaginally, there are studies that have published the benefits of performing an episiotomy for reducing the risk of high-grade perineal tears [11,12]. However, it is increasingly important to individualize and select the indication for an episiotomy, including in instrumental deliveries, by knowing the distension of the pelvic floor [11,13], even at the risk of tearing, which is often only minor [14].

It is clear that pelvic floor morbidity associated with vaginal delivery would increase with a decrease in the rate of caesarean sections. However, the increased maternal and perinatal morbidity and mortality associated with this surgical procedure (hemorrhage, infection, damage to neighboring organs, and neonatal distress) has led institutions such as the WHO recommend a reduction in the rate of caesarean sections [15], among other morbimortality reasons, as caesarean sections increase the risks of future caesarean sections in the same patient.

The aim of this study is to analyze the distribution of maternal−perinatal variables in the group of postpartum patients with third and fourth degree perineal tears in our reference population compared with patients without injury or with a mild degree of injury, as well as to determine the distribution and association of the maternal−perinatal variables most associated with third and fourth degree perineal tear events. The main hypothesis of this study is that the identification of maternal and perinatal risk factors can help to minimize third and fourth degree perineal tears during childbirth.

## 2. Materials and Methods

An observational, ambispective study was conducted on a hospital-based cohort of patients whose deliveries (>22 weeks gestational age and/or >500 g at birth) were attended to in the obstetrics service of the HGUGM (Hospital General Universitario Gregorio Marañón de Madrid) during the period from January 2010 to April 2017. Over the years, a prospective record was made in the database, although the analysis of the data was retrospective. The diagnosis of second, third, and fourth degree tears was made by obstetric physicians, even when these patients were initially attended to by midwifery. Records of third and fourth degree tears were made by the obstetrical doctors who were responsible for the repair of the tear, after the surgical process in the delivery room, once it had been completed.

We do not have the follow-up data at the moment for severe perineal tears. 

Exclusion criteria include caesarean delivery, fetal exitus, and multiple gestations.

The patients were divided into two groups: group I, those with a third and fourth degree tears, and group II, the rest of the patients, including those with less severe tears (first and second degree tears).

In both groups of patients, the maternal (age, defined as the maternal age at the time of delivery; gestational age, the number of weeks of gestation at the time of delivery; and parity, the number of previous deliveries of the patient), obstetric (spontaneous/induced delivery, with spontaneous defined as those in which no medical procedure was used to initiate delivery, and induced as those in which medical treatment was used to initiate delivery; epidural anesthesia/no anesthesia, considering epidural anesthesia as those procedures in which an anesthetic was applied through lumbar puncture in the spinal region, and non-anesthesia as the absence of the application of anesthetic; eutocic/instrumental delivery, with instrumental defined as those procedures in which forceps, vacuum, or spatulas were used vs. those that did not require instrumental procedures; performance of episiotomy, the performance of a perineal cut in the third stage of labor to facilitate its progression; and intrapartum pH, the measurement of pH in the fetal calyx), and neonatal (weight of the newborns, Apgar test values at 1 and 5 min, etc.) variables were studied.

The data collection method was carried out by the delivery room staff, recording all of the data of the different patient variables in a digitalized system, which was subsequently transferred to an Excel spreadsheet for dissociation and analysis, using the Microsoft Office Excel database, version 2019 (Microsoft, Redmond, WA, USA). The statistical analysis was performed with SPSS version 25 (IBM Corp. Released 2017. IBM SPSS Statistics for Windows, Version 25.0. IBM Corp., Armonk, NY, USA). For each group, the descriptive parameters for the mean, standard deviation, and 95% confidence interval were calculated for all quantitative variables, and for the qualitative variables, the relative percentage frequencies were calculated. Hypothesis testing for the comparison of the results of variables in each group was carried out by applying the tests according to the type of variable and its distribution. For the independent calculation of the magnitude of effect of each variable, a predictive/explanatory analysis of the uni- and multi-variate logistic regression was carried out. After the first univariate analysis, those variables clinically relevant and/or with a *p* < 0.2 were taken into consideration for inclusion in the multivariate analysis in order to assess the magnitude of association weighted by all of the variables included. A *p* < 0.05 was defined for statistical significance.

## 3. Results

**Figure 1:**
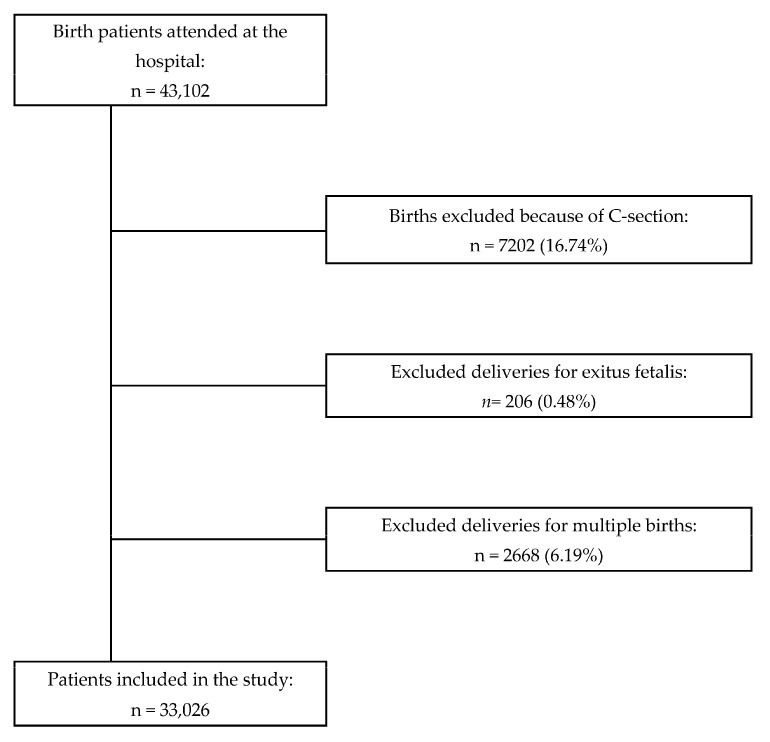
Flow chart for the inclusion/exclusion of the patients in the study.

During the study period 43,025 patients were delivered in our centre. The flow diagram of the patients included in the study can be seen in Figure 1.

Of the 33,026 patients, 301 (0.91%) were in group I and 32,725 were in group II (99.09%).

Table 1 presents the distribution of the study variables for all of the patients and a comparative analysis of these variables according to the study group, adding the statistical significance of this analysis according to the appropriate hypothesis test. It should be noted that the mean maternal age was 32 ± 5.74 years, with the majority of patients aged 30–35 years. For all of the patients, 52.3% were nulliparous, with this percentage being significantly higher in the severe tearing group (79.1% vs. 48.2%; *p* < 0.001). As for the induction of labor, there were 84 patients (27.9% vs. 21.4%); *p* = 0.046), a slight increase. Epidural or spinal regional anesthesia were significantly more frequent in group I compared with the rest (85.7% vs. 78.6%; *p* = 0.003). Episiotomy was performed in 18,025 (59%) of the cases, with a much higher incidence of severe tearing in 248 (82.4%) patients compared with the rest of the patients. The mean birth weight of the newborns in the study was 3237 ±483.94 g. The detailed results for these variables are shown in Table 1.

**Figure 2:**
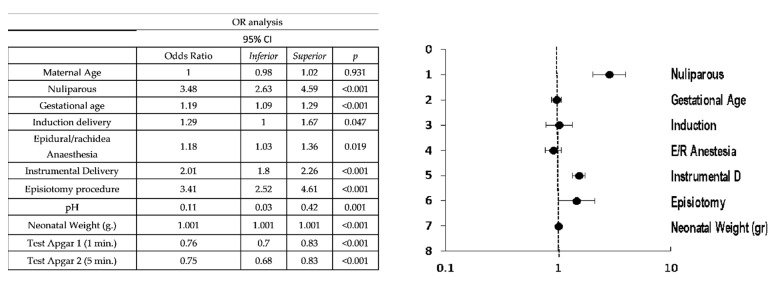
Comparison of risk factors for third and fourth degree perineal tears.

Figure 2 describes the degree of association according to the odds ratios of the study variables with the risk of third and fourth degree tears (group I). The variables most associated with third and fourth degree tears in the univariate analysis were, significantly, nulliparity (OR = 3.48; *p <* 0.001), instrumental delivery (OR = 2.01; *p <* 0.001), episiotomy (OR = 3.41; *p <* 0.001), and newborn weight (OR = 1.001/g; *p <* 0.001). In the multivariate analysis, primiparity (OR = 2.82; *p <* 0.001), instrumental delivery (OR = 1.52; *p <* 0.001), episiotomy (OR = 1.46; *p* = 0.045), and fetal weight (OR = 1.001/g; *p <* 0.001) were presented as risk factors for severe tearing (OR = 1.001/g; *p <* 0.001).

A more precise study was carried out in relation to the significant variables in the univariate analysis. In relation to the maternal variables, it is worth highlighting the increase in risk associated with each vaginal birth, both the nulliparity (OR = 3.40; *p* < 0.001) and for delivery after >38 weeks gestation (OR = 2.11; *p* = 0.002), while in the obstetric variables, induction of labor (OR = 1.29; *p* = 0.047); instrumentalization, in particular with forceps (OR = 4.51; *p* < 0.001); and the performance of an episiotomy in instrumental procedures (OR = 3.11; *p* < 0.001) are highlighted as those that most increase the risk of severe tearing. Regarding fetal variables, it should be noted that the weight of newborns increases the risk of developing third and fourth degree tears by approximately two times for every 500 g increase in weight from 3000 g, as shown in Table 2 and Table 3. 

## 4. Discussion

The results obtained from 33,026 women attended to between 2010 and 2017 show that, in a vaginal delivery, the percentage of third and fourth degree tears is 0.91%. In these cases, significantly higher percentages of primiparous, induced deliveries, epidural/rachidial anesthesia, instrumental delivery, and episiotomy were observed compared with the rest of the patients. Similarly, in group I, we found a significant increase in the mean maternal age, gestational age at delivery, and pH, with a decrease in the Apgar test values at 1 and 5 min, although the differences found were not significant. In line with these results, the multivariate analysis showed a significant association with third and fourth degree tearing for primiparity, gestational age, induction, type of anesthesia, instrumental delivery, episiotomy, and neonatal weight.

Regarding the incidence of perineal tears, different studies have established an incidence of third and fourth degree tears between 0.1% and 10.9% [3,4]. In the Euro-Peristat study [4], in which a large number of European countries participated, with a sample of almost 2 million women, the incidence of severe tearing in different countries is described with figures similar to ours, such as 1.8% in Germany, 3.2% in the UK, and 0.8% in France. Based on these data, our results for severe perineal tearing are a low incidence of 0.91%, with an *N* of 33,026 patients, in relation to other surrounding countries. However, in relation to the large differences found between the incidence ranges between countries, we note that there is no cause identified in the study for these differences. In fact, it highlights the importance of improving the assessment and reporting of perineal tears in each country, as it is possible that there are different criteria, even defined by the study itself, when diagnosing different tears. In fact, most fourth degree tears can be diagnosed as they require surgical repair of the rectal mucosa, whereas third degree tears may remain under or undiagnosed.

It is noteworthy that 79.1% of the women who had a third and fourth degree tear were primiparous. In the study by Maghoub et al. [16] with a similar *N*, 73.2% were primiparous. In fact, our study presents an OR = 3.48, *p* < 0.001, for nulliparity, compared with the OR = 3.50, *p* < 0.001, of Maghoub et al., so we can highlight that the biomechanical stress of parturition, which can produce micro rupture or rupture, decreases with parity.

The induction of labor is also associated with an increased risk of severe tearing. Wilson et al. [17] in a review with an *N* of more than 400,000 women, presented a very similar risk of third and fourth degree tears (OR = 1.08; *p* = 0.01) to the result presented in our study (OR = 1.29; *p* = 0.047).

In our study, among the patients with severe tearing, 52.5% had an instrumental delivery vs. 19.8% who had mild tearing. The analysis significantly quadruples the risk for forceps and spatula deliveries, with similar ORs, compared with a minor increase in risk associated with vacuum extraction (Table 2). Other studies also establish an increased risk in relation to instrumental procedures and third and fourth degree tears [18,19,20,21]. It is indisputable that, after correct application, the function of both forceps and spatulas is to increase the birth canal, with the risk of injury being to the pelvic floor in order to benefit the fetus. However, it is important to note that instrumental deliveries are mainly performed in patients with a dystocic delivery mechanics or by fetal indication for immediate extraction in cases where vaginal delivery can be performed. On the other hand, in instrumental vacuum extraction-type deliveries, the mechanism is only a tractor on the fetal head, without widening the diameter of the exit plane, which has less impact on the pelvic floor. This could explain why, despite the increased risk in vacuum extraction deliveries, the result was not significant, although other studies show vacuum extraction as a significant risk factor [22,23]. More specific studies that can establish the differences in risk for severe tearing between different instrumental procedures would be desirable.

In relation to episiotomy, we found a significantly higher percentage in the severe perineal tear group vs. the rest. We observed that, from the multivariate analysis, the magnitude of the effect decreased compared with the result obtained in the univariate analysis. In routine clinical practice, an episiotomy is performed prophylactically and there is clear controversy in the literature regarding its association with severe tears. For Gebuza et al. [11], a rate of 46.2% showed that a decreased episiotomy performance increased mild to moderate tears (first and second degree), while it may protect against severe tears. Other studies, however, show episiotomy to be a factor in severe tearing [16,24,25]. It should be added that, in order to further investigate the implication of this factor, other variables should be taken into consideration, such as the size and depth of the episiotomy, and whether the tear occurs in continuity with the incision line or outside of it.

Finally, it should be noted that a birth weight of over 3000 g doubles the risk of suffering a third and fourth degree tear for every additional 500 g, possibly due to the greater increase in biomechanical stress on the vulvar fork, as the newborns are larger, with higher head circumference diameters. Other studies also show that a birth weight greater than 3500 g has been established as a risk factor for severe tearing [11,23].

The study has limitations that are important to take into consideration when interpreting the data. Firstly, we consider that our reference center has an increased percentage of patients with a maternal pathology. Secondly, as mentioned above, there are other variables that were not the focus of this study, and that could provide additional information. Such is the case for variables such as ethnicity, BMI, weight gain, the existence of shoulder dystocia, and the characteristics of the episiotomy and of the tear itself. In addition, anal ultrasound in the delivery room has been reported in other studies as a useful tool in the more accurate diagnosis of third and fourth degree tears [26], although in our study, it was not included in the diagnosis of perineal tears.

In relation to the strengths of the study, although our results are not novel and support those obtained previously by the various studies already described in the discussion, one of our strengths is that, to our knowledge, it is the first to be carried out in our country and in a center with a large number of births over so many years. On the other hand, the methodology of being able to perform a multivariate analysis considering both maternal and neonatal variables, such as the type of instrumental delivery and its association, as well as the cut-off points for neonatal weight, stands out and obtained data from a hospital cohort of more than 40,000 deliveries, attended over more than 7 years, in a tertiary hospital in Madrid, one of the reference hospitals for obstetrics in Spain. As a result of the large sample size of our study, we found a statistical power of more than 80%, with which we were able to find significant associations (*p* < 0.05) in the differences found between the maternal-neonatal variables between the groups. Specifically, for the statistical significance found *p* = 0.03 after comparing the proportion of the different types of anesthesia found between the groups with our sample size, we reached a statistical power of 80%. 

## 5. Conclusions

The frequency of severe third and fourth degree perineal tears is low in the population of women who deliver vaginally—about 1 in 100 patients. The uni- and multi-variate regression analysis showed that primiparity, induction of labor, episiotomy, instrumental delivery with forceps or spatula, and a birth weight of 3000 g or more are significantly associated with an increased risk of third and fourth degree tears. Among these variables, episiotomy, instrumental delivery, and the birth weight of newborns are particularly relevant, all of which are modifiable factors in delivery care.

## Figures and Tables

**Table 1:** Characteristics of the study population. Group I includes third and fourth degree perineal tears and group II includes the rest of the patients. Instrumental delivery includes forceps, vacuums, and spatulas.

	Overall *n* = 33,026		Group I *n* = 301		Group II *n* = 32,725		
	Mean	SD	Mean	SD	Mean	SD	
	*n* (%)		*n* (%)		*n* (%)		*p*
**Maternal age at birth (Years)**	32	±5.74	32.36	±5.57	32.22	±5.79	<0.001
**Groups**							0.483
Under 25	3855 (11.7)		27 (9.0)		3828 (11.7)		
25 to 30	5743 (17.4)		57 (18.9)		5686 (17.4)		
30 to 35	11,335 (34.3)		112 (37.2)		11,223 (34.3)		
35 to 40	9700 (29.4)		84 (27.9)		9616 (29.4)		
>40	2327 (7.0)		21 (7.0)		2306 (7.0)		
N/A	66 (0.2)		0 (0)		66 (0.2)		
**Nulliparity**	16,019 (52.3)		238 (79.1)		15,781 (48,2)		<0.001
**Gestational age at delivery**	39.01	±1.78	38.99	±1.60	38.58	±2.42	<0.001
**Induced delivery**	7088 (23.1)		84 (27.9)		7004 (21.4)		0.046
**Type of anaesthesia**							0.003
None	2428 (7.3)		8 (2.7)		2420 (7.4)		
Perineal	2875 (8,7)		32 (10.6)		2843 (8.7)		
Epidural and/or spinal	25,319 (76.7)		258 (85.7)		25,061 (76,6)		
General	2404 (7.3)		3 (1.0)		2401 (7.3)		
**Instrumental delivery**	6643 (21.7)		158 (52.5)		6485 (19.8)		<0.001
**Episiotomy procedure at delivery**	18,025 (59.0)		248 (82.4)		17,777 (54.3)		<0.001
**Cord blood pH after delivery**	7.28	±0.76	7.29	±0.07	7.28	±0.08	<0.001
**Weight of newborn**	3237	±483.94	3,259	±471.82	3,130	±609.87	<0.001
**Test Apgar 1 (1 min.)**	8.75	±0.09	8.85	±0.92	8.55	±1.21	<0.001
**Test Apgar 2 (5 min.)**	9.68	±0.65	9.73	±0.80	9.55	±0.97	<0.001

**Table 2:** Adjusted odds ratio risk factors for third and fourth degree perineal tears.

		95% CI		
	Odds Ratio	*Inferior*	*Superior*	*p*
Maternal Age				
Nuliparous	2.82	2.02	3.94	<0.001
Gestational age	0.97	0.88	1.07	0.542
Induction delivery	1.02	0.78	1.33	0.895
Epidural/rachidea Anaesthesia	0.91	0.77	1.06	0.216
Instrumental Delivery	1.52	1.33	1.74	<0.001
Episiotomy procedure	1.46	1.01	2.11	0.045
pH	1.77	0.35	9.01	0.491
Neonatal Weight (g.)	1.001	1.001	1.001	<0.001
Test Apgar 1 (1 min.)	0.92	0.79	1.07	0.299
Test Apgar 2 (5 min.)	0.84	0.68	1.02	0.083

**Table 3:** Comparison of variables within relevant groups regarding risk of third and fourth degree perineal tears.

		95% CI		
	Odds Ratio	*Inferior*	*Superior*	*p*
**Nulliparity**	3.48	2.65	4.62	<0.001
**Gestation at term (>38 weeks)**	2.11	1.32	3.36	0.002
**Induction delivery**	1.29	1	1.67	0.047
**Instrumental Delivery**				
Forceps	4.51	3.57	5.7	<0.001
Vacuum	1.41	0.69	2.88	0.345
Spatula	4.35	2.02	9.37	<0.001
**Eutocic Delivery**				
Episiotomy procedure	1.98	1.4	2.79	<0.001
**Episiotomy procedure**				
Instrumental delivery	3.11	2.40	4.02	<0.001
**Fetal Weight**				
>3000 g	2.41	1.73	3.37	<0.001
>3500 g	1.97	1.56	2.47	<0.001
>4000 g	2.17	1.46	3.24	<0.001

## Data Availability

Data from this study are available at the obstetrics service of the Hospital General Universitario Gregorio Marañón in Madrid and will be made available upon request.

## Abbreviations

OR	Odds ratio
C-Section	Caesarean birth
UI	Urinary incontinence
FI	Fecal incontinence
AI	Anal incontinence
pH	Hydrogen potential
CI	Confidence interval
I2	I squared
PGE1	Prostaglandin E1
PGE2	Prostaglandin E2
WHO	World Health Organization
HGUGM	Gregorio Marañón General University Hospital in Madrid
